# Environmental sustainability in endodontics. A life cycle assessment (LCA) of a root canal treatment procedure

**DOI:** 10.1186/s12903-020-01337-7

**Published:** 2020-12-01

**Authors:** Brett Duane, Linnea Borglin, Stephanie Pekarski, Sophie Saget, Henry Fergus Duncan

**Affiliations:** 1 Trinity College Dublin, School of Dentistry Lincoln Place Dublin, IE 2, Dublin, D02 F859 Ireland; 2 Faculty of Odontology Malmo, Malmo Universitet, Skåne, Sweden; 3 Department of Botany Dublin, Trinity College Dublin, Dublin, Ireland; 4 Division of Restorative Dentistry & Periodontology, Trinity College Dublin, Dublin Dental University Hospital, University of Dublin, Lincoln Place Dublin 2, Dublin, Ireland

**Keywords:** Sustainability, Environment, LCA, Life cycle analysis, Endodontics, RCTx, Isopropyl alcohol, Dental gown, Single use instruments

## Abstract

**Background:**

To analyse via life cycle analysis (LCA) the global resource use and environmental output of the endodontic procedure.

**Methodology:**

An LCA was conducted to measure the life cycle of a standard/routine two-visit RCT. The LCA was conducted according to the International Organization of Standardization guidelines; ISO 14040:2006. All clinical elements of an endodontic treatment (RCT) were input into OpenLCA software using process and flows from the ecoinvent database. Travel to and from the dental clinic was not included. Environmental outputs included abiotic depletion, acidification, freshwater ecotoxicity/eutrophication, human toxicity, cancer/non cancer effects, ionizing radiation, global warming, marine eutrophication, ozone depletion, photochemical ozone formation and terrestrial eutrophication.

**Results:**

An RCT procedure contributes 4.9 kg of carbon dioxide equivalent (CO2 eq) emissions. This is the equivalent of a 30 km drive in a small car. The main 5 contributors were dental clothing followed by surface disinfection (isopropanol), disposable bib (paper and plastic), single-use stainless steel instruments and electricity use. Although this LCA has illustrated the effect endodontic treatment has on the environment, there are a number of limitations that may influence the validity of the results.

**Conclusions:**

The endodontic team need to consider how they can reduce the environmental burden of endodontic care. One immediate area of focus might be to consider alternatives to isopropyl alcohol, and look at paper, single use instrument and electricity use. Longer term, research into environmentally-friendly medicaments should continue to investigate the replacement of current cytotoxic gold standards with possible natural alternatives. Minimally invasive regenerative endodontics techniques designed to stimulate repair or regeneration of damaged pulp tissue may also be one way of improving the environmental impact of an RCT.

## Background

Global sustainability is the number one public health issue. A sustainable world must meet the needs of the present without compromising the ability of future generations to meet their own needs [1]. Currently, the delivery of healthcare is not sustainable. Healthcare systems are harming both the public and the planet with UK healthcare accounting for around 4% of the total national carbon footprint (SDU 2016) and additional harm caused by the release of healthcare associated travel emissions resulting in a loss of 614,000 disability-adjusted life years (DALY) in the US annually [2, 3]., Most countries worldwide have signed up to the Paris agreement which makes it mandatory for countries to reduce their net carbon emissions to zero by 2050–2100 and stop global temperatures rising more than two degrees Celsius [4].

Healthcare consumes significant energy, requires travel, and as an industry procures a number of different types of reusable and disposable instruments, and produces significant waste.

There is considerable debate relating to the damages caused by single use plastic [5, 6] the production of paper is also harmful being the main contributor to deforestation, having a negative effect on water systems and accounting for 12–18% of world-wide GHG emissions [7, 8].

Not only are the products we purchase important from an environmental perspective but so is the way we manage their disposal. According to Cherubini et al. [9] the use of landfills are a poor strategy in terms of waste management, due to the release of methane (CH4) and other landfill gases into the atmosphere. The process of incineration is however controversial [10]. Using appropriate measures such as filtering the released gas greatly reduces the amount of toxins released from the plant. As a result, the major by-products are CO_2_ and water [11]. However if, during the burning of waste, incomplete combustion occurs, hazardous and environmentally dangerous organic hydrocarbons may be released, many of them being carcinogenic and mutagenic [12].

With dentistry, patient travel and staff travel (for both work purposes and to commute) make up around 60 % of the total dental carbon footprint. Energy is another contributor [13]. The third contributor is the items procured by a dental practice. Dental care uses large amounts of paper, plastic and stainless steel products, both disposable and reusable. There is a growing realisation that sustainability is not just about carbon emissions but also about the type of materials we buy, the waste we produce and our impact on biodiversity [14–16].,

Life cycle assessment (LCA) is a technique used to understand and assess the environmental impact of a product system or process. The life cycle of a product includes stages including; raw material acquisition, the production process, disposal and transportation [17]. By using an LCA it is possible to evaluate the potential environmental impact that different dental procedures will have across the different impact categories. According to the FDI the need for research on improvement within sustainability in dentistry should be promoted [18]. From our understanding to date, life cycle analysis has only been used in only one paper in dentistry [19]. As a result, there is a need to perform life cycle analysis across dentistry to better understand resource usage and from an environmental perspective, the impact of the products and systems we use.

The discipline of endodontics encompasses a range of techniques aimed at preserving the vitality of dental pulp or preventing or eliminating apical disease [20]. The most common endodontic procedure is root canal treatment (RCT), in which the inflamed or necrotic pulp is removed and replaced with an inert material, thereby preserving the tooth. During an RCT, the dentist uses a large number of single or limited-use instruments (root canal instruments) as well as a range of other consumables including water, energy, paper, medicinal products and medical devices. The procedure is intricate and technically demanding, requiring prolonged and often multiple appointments to carry out the RCT to a high standard. Success of RCT is measured by the absence of signs and symptoms of apical infection and relies on the effective elimination of microorganisms from the root canal system [21]. Removal of the root canal infection is achieved by chemo-mechanical disinfection utilising root canal instruments in combination with disinfecting agents such as sodium hypochlorite (NaOCl) solution (0.5–5%), ethylenediaminetetraacetic acid (EDTA) and chlorhexidine [22]. It is proposed that the combination of relatively high resource usage and the time spent within the dental surgery, has underestimated the actual environmental footprint. The international community is unaware of which specific steps of an RCT would threaten the environment. The aim of this study was to assess and quantify the life cycle of an RCT.

## Methods

### Life cycle assessment (LCA)

An LCA was conducted to measure the life cycle of a standard/routine two-visit RCT, at the Faculty of Dentistry, Malmö University, Sweden. The equipment and products analysed were those used as part of standard kits issued for treatments at the faculty. The results were used to model the natural resources required and the pollutants emitted to quantify the environmental consequences of each of the components of RCT. The LCA was conducted according to the International Organization of Standardization guidelines; ISO 14040:2006. *OpenLCA* is a free, life cycle assessment software and was the chosen software for this study. The databases *openLCA LCIA methods v1.5.7* and v2 were chosen, which include an extensive collection of life cycle impact assessment methods, with some of them being country-specific [23]. The database ecoinvent version 3 was used to access activity datasets that form the basis to the system modelling http://v35.ecoquery.ecoinvent.org/Search/Index.

### Goal, scope and system boundaries

The goal of this LCA was to evaluate the environmental impact of a routine two-visit RCT. To conduct as thorough a study as possible, as many aspects of the procedure as practically possible were included.

**Fig. 1:**
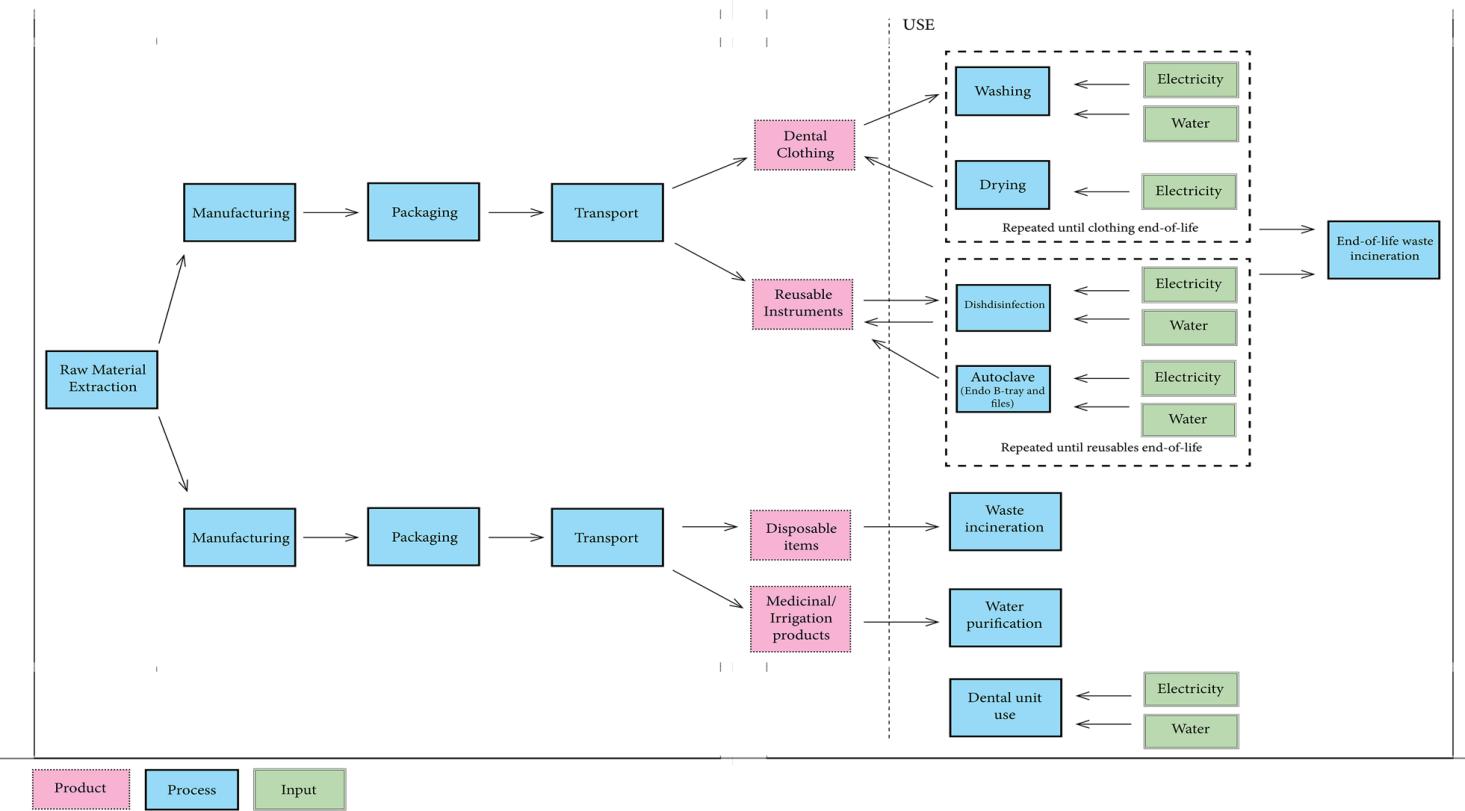
System boundaries

For this study, the functional unit was defined as one RCT procedure. The production, use, disinfection, sterilisation and disposal of all disposable and single use instruments; production, washing and drying and disposal of dental clothing; water and energy use associated with the disinfection and sterilisation of instruments, the use and disinfection of the dental unit and the hand washing of the dentist were all included. For the purpose of this study, the construction of the faculty building and the production of large machines such as the dishwashers, the dental unit and other electrical appliances (e.g. computers) were excluded. Staff and patient travel were also excluded. The system boundaries are illustrated in Fig. 1. This illustrates the cradle-to-grave aspects of disposable and reusable products and includes the manufacturing, transportation, use and waste management of each product.

### List of assumptions

In order to facilitate comparison a number of assumptions were made within this LCA including;

1. The RCT procedure was completed in 2 sessions (patient visits).
2. All products coming from Sweden (< 30 km) were transported in a small lorry.
3. Products with a European origin (> 30 km) were transported in a large lorry.
4. The products produced outside of Europe were first transported with a large lorry to the closest port, then by cargo ship to Malmö port and finally by small lorry to the distributor.
5. All land transport is calculated based on European transport.
6. The packaging was assumed to be cardboard, weighing 10% of each product.
7. The dentist and nurse use one set of clothes per procedure.
8. During a two visit RCT procedure, the dental unit is cleaned a total of four times, twice per session (2 sessions). 100 ml of surface disinfection is used, along with four paper towels per clean. For this analysis the unit was only cleaned after the session.
9. Each time the dentist and nurse wash their hands, one litre of water, 10 ml of hand soap and 5 ml of hand disinfection is used.
10. 12 trays are loaded in the dishwasher and autoclave during each standard cycle.
11. The autoclave consumes six litres of water during each cycle.
12. All disposable products enter the general waste stream and were not classified as hazardous waste.
13. At the end of their lifetime all stainless steel and nickel titanium (NiTi) products were either recycled or the metal recovered after incineration.

### Life cycle inventory

#### Data collection

Primary data collection was done at the faculty. An inventory of each kit was created, and each disposable and reusable item was weighed using a Gibertini Europe 600 scale to two decimals (± 0.02) [24]. When possible, ten of each item were weighed to calculate the average. The lifetime of each reusable product was based on conservative estimates provided by the dental faculty staff in both Malmö University and Dublin Dental University Hospital (DDUH). All instruments were classified according to their material composition. See Additional files 1 and 2: Appendix 1 and 2 for detailed lists on the standard composition of an endodontic kit. The process of disinfecting and sterilising each kit was directly observed. Information on the laundry process of the dental scrubs was obtained from the relevant faculty staff. Data on the type of electricity and waste disposal was acquired from the faculty’s facility manager.

#### Transport

Transport distances were based on the manufacturing locations of each product and the location of the local distributors in Malmö, Sweden. Unfortunately, the manufacturing locations could not be sourced for some products. As a result, they were assumed to originate from the locations of other similar products. The distance between the local distributor and the faculty was excluded since the distance is minor and would likely result in negligible differences in CO_2_ eq emissions. Transport distances were estimated using *Searates* (www.searates.com).

### Dentist preparation

The dentist and nurse wear a set of dental clothing which consists of a shirt, trousers and a coat. The sets are loaded into a washing machine and dryer with a capacity of 25 sets per cycle. The water and energy consumption of the washing machine and dryer are summarized in Table 1. Before meeting the patient, the dentist washes and disinfects their hands.

**Table 1:** Energy and water consumption values for the machines used according to the manufacturers

Machine	Brand	Power (kW)	Time (mins)	Energy (kWh)	Water (litres)
Dishwasher	KEN IWD 2314	1.00	50.00	0.83	55
Autoclave (with built in compressor)	Matachana SC500	21.00	57.00	19.95	6^**^
Central compressor	Kaeser SM15T	9.00	see Table 2	see Table 2	0
Washing machine	Electrolux	^*^	50.00	0.4–1.0	197
Dryer	Electrolux	24.00	15.00	6.00	0
Intraoral Imaging (per image)	Planmeca ProX	0.5600	0.0003	0.0002	0
DAC UNIVERSAL	Nitram	11.0000	12.0000	2.2000	0.3334

Assorted units in kilowatt (kW), minutes and litres

### The dental unit

The energy and water consumption of the dental unit was only calculated for the duration of the procedure. Table 2 describes the average procedure times and the power and water consumption associated with the unit.

**Table 2:** A summary of the average procedure time, energy and water usage, and the estimated usage time of instruments during each procedure. Units in minutes and kilowatt hours. Adapted from Duane et al. [25]

Dental unit use by procedure type	
Duration (mins)	180
Water Usage (ml)	500

Machines used and rating	Equipment usage Per appointment type (mins)	kWh endo
Dental unit motor (400 W)	3	1.2
Dental light (30-40 W)	180	7.2
Unit screen (20-30 W)	0	0
Instrument light (2,5 W)	45	0.1125
Suction (9 kW)	45	1.875
Machines operated by compressor (9 kW)	60	9
		Total power consumed
		19.3875

### Use

All disposable products were discarded after a single use.

**Fig. 2:**
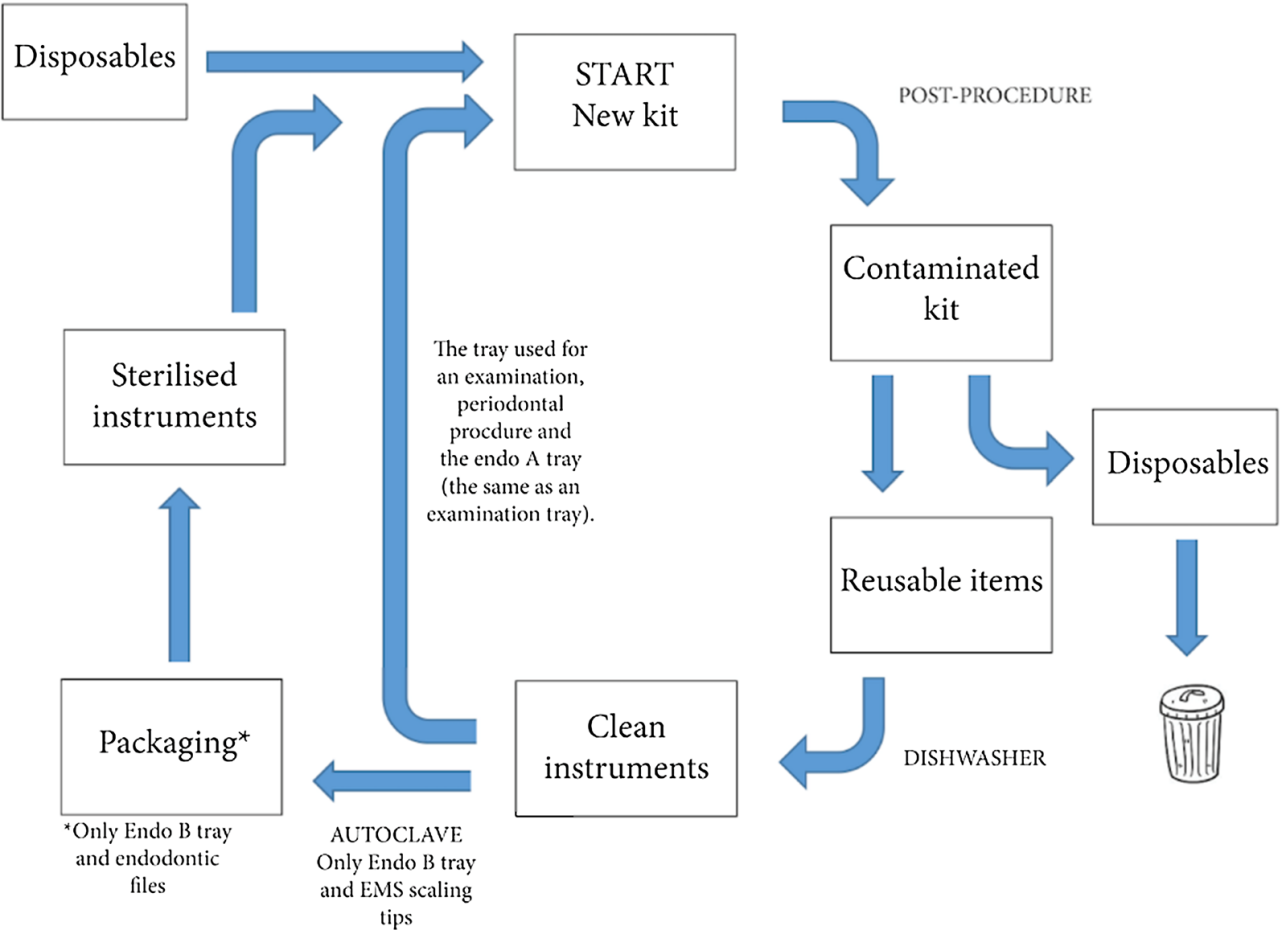
A flow chart describing the cleaning and sterilisation process of the dental kits

Most reusable stainless steel products were disinfected in the dishwasher after use, with the exception of the instruments on the endodontic B tray, which is autoclaved and packaged in sterile bags. The procedure is described in Fig. 2 below. All reusable products are washed in the KEN IWD 2311 dishwasher. Six endodontic kits can be loaded during each cycle. The steam steriliser used is the Matachana SC500. The handpieces are washed and sterilised in the Nitram DAC Universal, which has a capacity of six handpieces. Data on energy and water consumption were obtained directly from the distributor. The extended burs are cleaned in an ultrasonic cleaner, prior to disinfection. However, due to insufficient information on the energy use of the ultrasonic cleaner, this was excluded from the scope of the study. Any servicing or repairs of instruments such as the handpieces were not included in the study analysis.

### Average procedure times

The total procedure time for an RCT was calculated as being three hours, as is the standard time for an RCT in Malmo. This was divided into two separate ninety minute sessions.

### Average consumables used during root canal treatment

Within Malmö University stainless steel endodontic K-files larger than International Organization for Standardization (ISO) size 20 are used five times, while stainless steel K-files below ISO 20 and reciprocating NiTi Wave One Gold® files (Dentsply Sirona, Ballaigues, Switzerland) are disposed of after a single-use. This was used as the basis for our assumptions. A standard set-up for an RCT procedure in Malmö included hand files (ISO size 10–60), NiTi files (3 WaveOne® Gold), a lentulo-needle and finger spreaders size B and C (Additional file 1: Appendix 1). The hand files were used for initial negotiation and apical sizing, while the NiTi files were used first for coronal shaping before moving to apical shaping and blending of the taper. As a result we assumed that all these files would be used within an average RCT procedure.

### Energy and water consumption

The energy consumption (kWh) for the electrical appliances was estimated by using average procedure times or the total running time for each standard program of the dishwasher, autoclave, washing machine and dryer. Other programs (such as the autoclave tests) were not taken into consideration and excluded from the study. The amount of water consumed during the endodontic procedure based on instrument usage (e.g. handpiece). Patient drinking water was excluded from this study.

### Disposal/end of life

The waste is disposed in a container that is emptied three times a week and transported to a recycling area in Malmö where it is incinerated. The energy released from waste incineration is used for district heating. The distance transported for this process was excluded from this study.

### Life cycle impact assessment

All data was classified and entered into the program *openLCA* for the LCIA. The inventory data can be seen in Table 3.

**Table 3:** Inventory data used for the life cycle inventory of an RCTx

Material/process	Product examples	Amount	LCI database	Database process name
Steel	Endodontic files, Dental explorer, pocket probe, carver, tray	25.78 g	ecoinvent v3.5	casting, steel, lost-wax | casting, steel, lost-wax | Cutoff, U - RoW
Isopropanol	Surface disinfection	180.00 g	ecoinvent v3.5	isopropanol production | isopropanol | Cutoff, U - RER
Tissue Paper	Paper towels, disposable bib, face mask	73.66 g	ecoinvent v3.5	market for tissue paper | tissue paper | Cutoff, U - GLO
Textile, woven cotton	Clothing, non-woven sponges	0.32 g	ecoinvent v3.5	market for textile, woven cotton | textile, woven cotton | Cutoff, U - GLO
Cotton fibre	Cotton pellets, non-woven sponges	1.82 g	ecoinvent v3.5	market for cotton fibre | cotton fibre | Cutoff, U - GLO
Electricity	Unit use, laundry, dishwasher	31.01 kWh	ecoinvent v3.5	electricity production, nuclear, pressure water reactor | electricity, high voltage | Cutoff, U - SE
Water	Hand washing, laundry, dishwashing	24.67 L	ecoinvent v3.5	market for tap water | tap water | Cutoff, U - Europe without Switzerland
Kraft paper	Paper points	0.18 g	ecoinvent v3.5	market for kraft paper, unbleached | kraft paper, unbleached | Cutoff, U - GLO
Iodine	Iodine solution	2.00 g	ecoinvent v3.5	market for iodine | iodine | Cutoff, U - GLO
Sodium Hypochlorite	Sodium Hypochlorite	91.00 g	ecoinvent v3.5	market for sodium hypochlorite, without water, in 15% solution state | sodium hypochlorite, without water, in 15% solution state | Cutoff, U - RER
EDTA	EDTA	6.00 g	ecoinvent v3.5	market for EDTA, ethylenediaminetetraacetic acid | EDTA, ethylenediaminetetraacetic acid | Cutoff, U - GLO
Zinc oxide	Temporary filling	4.00 g	ecoinvent v3.5	market for zinc oxide | zinc oxide | Cutoff, U - GLO
Rubber seal	Gutta perka, Rubber dam	9.38 g	ecoinvent v3.5	market for seal, natural rubber based | seal, natural rubber based | Cutoff, U - GLO
Epoxy resin	Sealer	2.00 g	ecoinvent v3.5	market for epoxy resin, liquid | epoxy resin, liquid | Cutoff, U - RER
Hydrogen peroxide	Hydrogen peroxide	2.00 g	ecoinvent v3.5	market for hydrogen peroxide, without water, in 50% solution state | hydrogen peroxide, without water, in 50% solution state | Cutoff, U - RER
Quicklime	Calcium hydroxide	0.67 g	ecoinvent v3.5	market for quicklime, milled, packed | quicklime, milled, packed | Cutoff, U - RER
Glass	Glass mixing tray, dappen dish	0.46 g	ecoinvent v3.5	flat glass production, uncoated | flat glass, uncoated | Cutoff, U - RER
Soap	Hand soap, detergents	119.90 g	ecoinvent v3.5	market for soap | soap | Cutoff, U - GLO
Cardboard	Cardboard packaging	297.12 g	ecoinvent v3.5	corrugated board box production | corrugated board box | Cutoff, U - RER
Nitrile	Dentist gloves	28.40 g	ecoinvent v3.5	market for acrylonitrile | acrylonitrile | Cutoff, U - GLO
Ethanol	Hand disinfection	75.00 g	ecoinvent v3.5	ethylene hydration | ethanol, without water, in 99.7% solution state, from ethylene | Cutoff, U - RER
Polypropylene	Plastic cup, evacuation tip adaptor	17.13 g	ecoinvent v3.5	polypropylene production, granulate | polypropylene, granulate | Cutoff, U - RER
Plastic film	Disposable bib, sterile bags	91.84 g	ecoinvent v3.5	market for packaging film, low density polyethylene | packaging film, low density polyethylene | Cutoff, U - GLO
Polyethylene	Evacuation tip,	57.79 g	ecoinvent v3.5	market for polyethylene terephthalate, granulate, bottle grade | polyethylene terephthalate, granulate, bottle grade | Cutoff, U - GLO
Electric motor	Handpiece, WaveOne, Apex localisator	0.64 g	ecoinvent v3.5	market for electric motor, for electric scooter | electric motor, for electric scooter | Cutoff, U - GLO
Electronic waste	Handpiece, WaveOne, Apex localisator	− 0.64 g	ecoinvent v3.5	Waste/ecopoints 97, CH
Emissions to air	Surface disinfection, hand disinfection	255.00 g	ecoinvent v3.5	Emission to air/high population density
Steel waste	Endodontic files, Dental explorer, pocket probe,	− 25.78 g	ecoinvent v3.5	Waste, unspecified
Waste Incineration	All other waste	630.29 g	ecoinvent v3.5	market for municipal solid waste | municipal solid waste | Cutoff, U - SE
Wastewater	All wastewater and liquids	− 24.85 L	ecoinvent v3.5	treatment of wastewater, from residence, capacity 1.1E10l/year | wastewater, from residence | Cutoff, U - RoW
Transport	Small lorry	2.60 km	ecoinvent v3.5	market for transport, freight, lorry 3.5-7.5 metric ton, EURO6 | transport, freight, lorry 3.5-7.5 metric ton, EURO6 | Cutoff, U - RER
	Large lorry	132.32 km	ecoinvent v3.5	market for transport, freight, lorry 16-32 metric ton, EURO6 | transport, freight, lorry 16-32 metric ton, EURO6 | Cutoff, U - RER
	Sea freight	433.65 km	ecoinvent v3.5	market for transport, freight, sea, transoceanic ship | transport, freight, sea, transoceanic ship | Cutoff, U - GLO

## Results

The main results of this Life Cycle Analysis can be seen in Table 4.

**Table 4:** Life cycle analysis results

Impact Description	Unit	Impact Quantity
Resource use, energy carriers	MJ	236.5688
Resource use, minerals and metals	kg Sb eq	1.78E−05
Acidification	molc H+ eq	0.07761
Freshwater ecotoxicity	CTUe	7.9846
Freshwater eutrophication	kg P eq	0.00174
Human toxicity, cancer effects	CTUh	1.95E-07
Human toxicity, non-cancer effects	CTUh	1.07E-06
Ionizing radiation HH	kBq U235 eq	13.39523
Climate Change	kg CO2 eq	4.90766
Marine eutrophication	kg N eq	0.0083
Ozone depletion	kg CFC-11 eq	1.10E-06
Photochemical ozone formation	kg NMVOC eq	0.12424
Terrestrial eutrophication	molc N eq	0.0554
Land use	Pt	270.9849
Respiratory inorganics	disease inc.	2.87E-07
Water scarcity	m3 depriv.	4.64765

An RCT procedure contributes 4.9 kg of carbon dioxide equivalent emissions. This is the equivalent of a 30 km drive in a small car (Mapmyemissions.com/home).

**Fig. 3:**
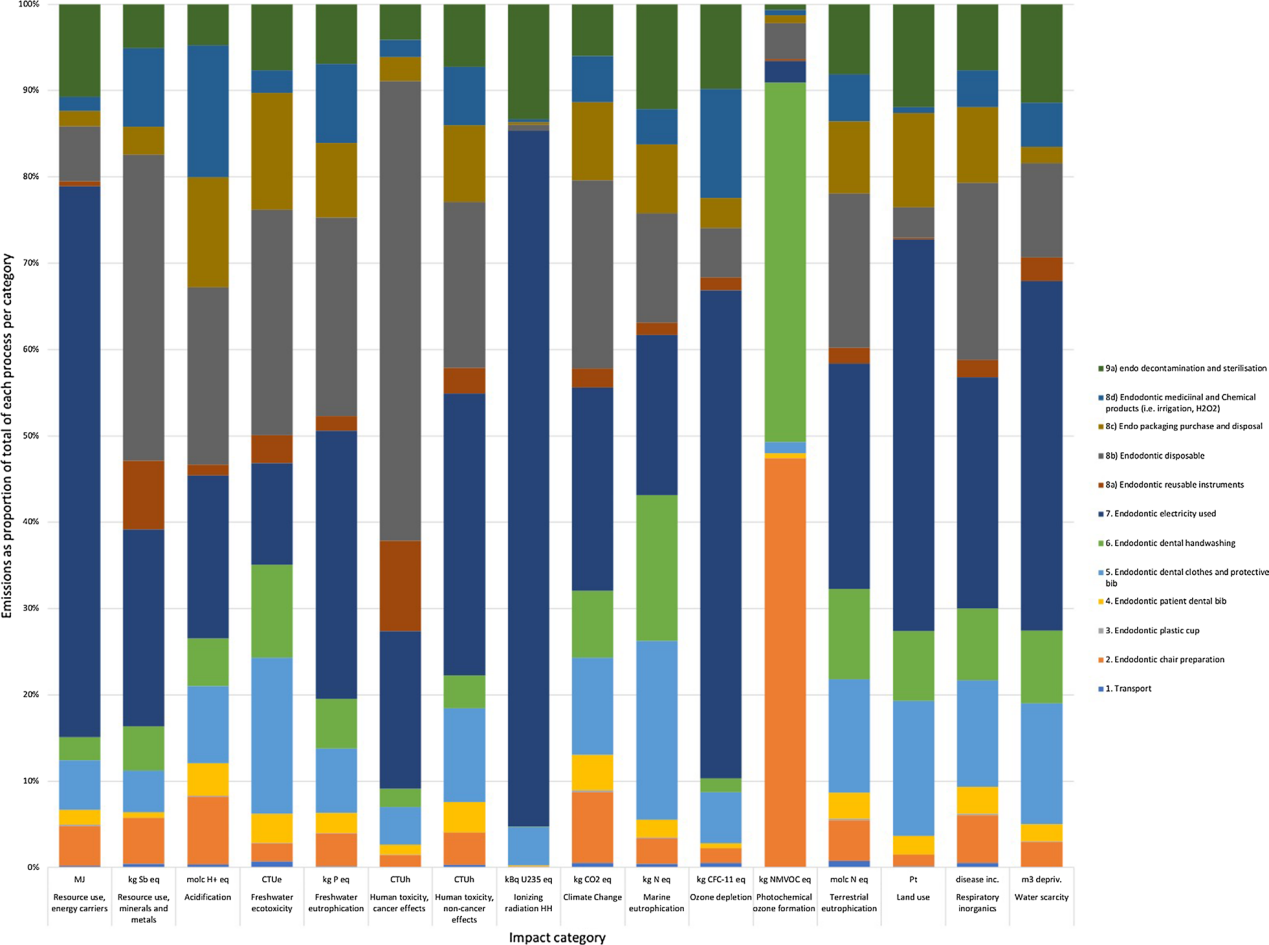
Life Cycle Assessment contributing elements for each process within an RCT

**Fig. 4:**
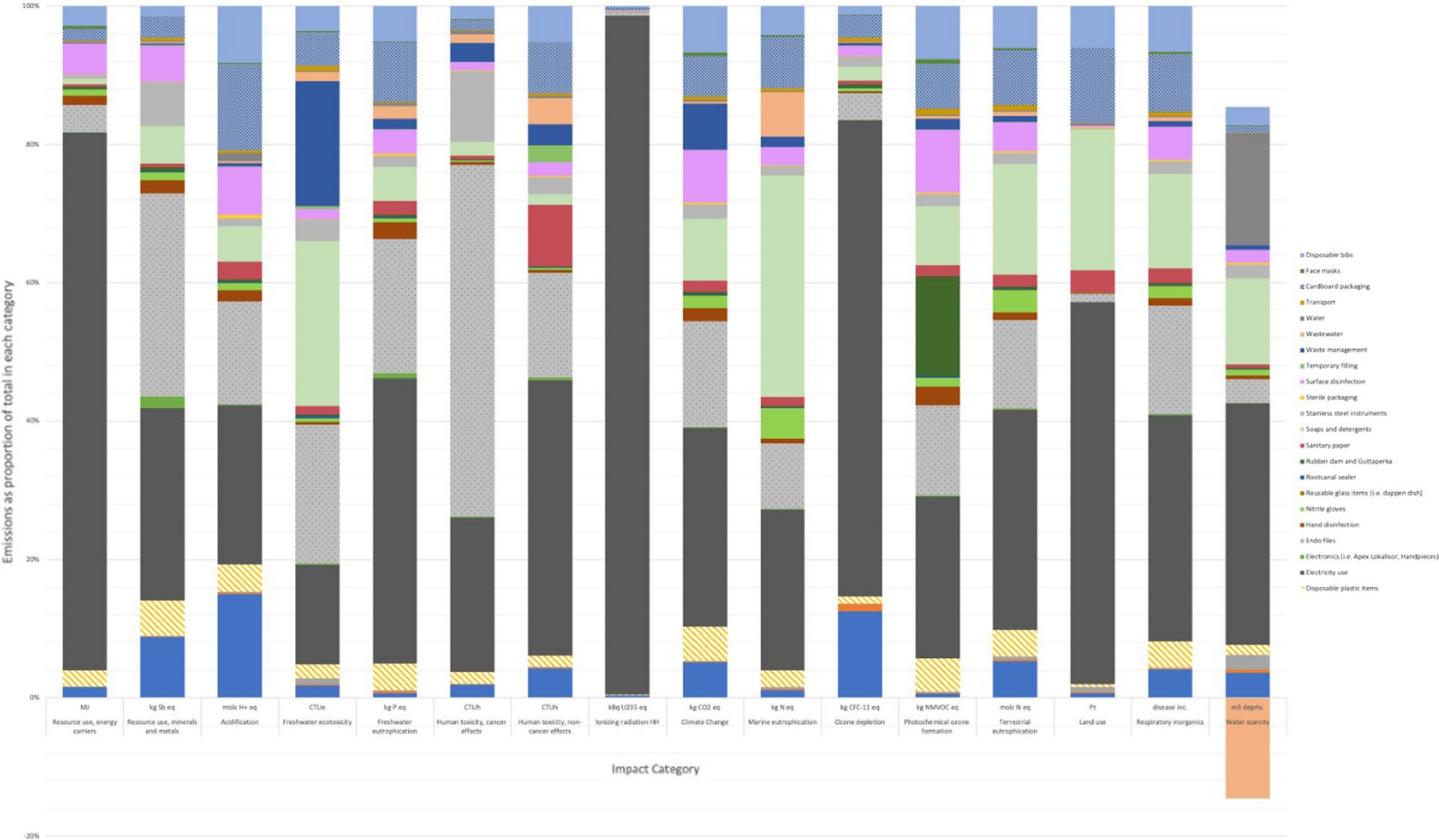
Contributing elements for materials used in an RCT

The environmental impact of an RCT depends on the impact category concerned. Figures 3 and 4 show that the most significant contributor to GWP within endodontics is the use of electricity and the impact from using dental instruments. Dental clothing contributes significantly to ozone depletion. This harm is shown in environmental impacts such as resource use, acidification, fresh water ecotoxcity/eutrophication, human toxicity. The transportation of endodontic goods contributed strongly to environmental harm in most areas.

From a global warming potential, the main contributors were electricity followed by single-use stainless steel instruments, soaps and detergent with a relatively similar contribution from surface disinfection, waste management, cardboard packaging, and disposable bib.

## Discussion

Recent studies on sustainability within dentistry have focused on the overall carbon footprint of the dental service and considered travel, procurement and building energy [25, 26]., A Public Health England (PHE) study [13] calculated the carbon footprint of an endodontic procedure to be 23.3 kg. The PHE figure is higher than our result (4.9 kg), in part because our study did not include patient travel, but also because we performed a more detailed bottom-up analysis of every medicament, and instrument used, rather than a simple top down approach based on financial data, and surgery time, and energy.

The aim of the current study was to use LCA to understand which elements of a standard RCT procedure have the largest potential environmental impact. With raised awareness about climate change the importance of all medical and dental sectors to do their part is increasing. By quantifying the potential environmental impacts, including the global warming potential resulting from a dental procedure, appropriate measures can be taken to reduce different parts of the procedure without compromising patient safety. Although this LCA has illustrated the effect RCT has on the environment, there are a number of limitations that may influence the validity of the results. The lack of freely available life cycle impact assessment (LCIA) data increases the uncertainty of the results. For manufactured products such as sanitary paper, medicinal and botanical ingredients as well as surgical and medical instruments, the analysis was based on US not European data. The actual LCA data could differ if the location of manufacture was not the US, especially as regulations often differ between countries (e.g. China [27]).

In order to clearly define the scope of the study additional assumptions were made. It is assumed that an RCT procedure was completed in two sessions as this represents the most common time frame for RCT completion [28, 29]. However, the actual number of sessions needed and as a result the materials used are dependent on the complexity of treatment including tooth-related, dentist-related and patient-related factors, all of which could result in less or significantly more than two appointments being needed to complete the treatment. This study assumes that the dentist works with a dental nurse, as in many countries a dental nurse is an integral part of the dental procedure. In the UK dental care professionals should be supported when treating a patient, which can be interpreted as requiring a dental nurse chairside (CQC 2019).

The differences in paper use, processes for wiping chair and varying amounts of water used between people and in diverse countries could significantly influence the LCA calculation. The assumption that 12 trays are loaded in the dishwasher and in the autoclave during each standard program could also differ from reality. The maximum load of the dishwasher in this setting is 12 trays and it was assumed to be the same for the autoclave. It is unlikely that the dishwasher and the autoclave are always run filled to maximum capacity. The water consumption associated with each autoclave cycle is also an estimation, since no information was obtained through direct contact with the company. Both these factors could affect the potential acidification, marine eutrophication and terrestrial eutrophication due to an increase in wastewater treatment. The defined lifespan of the reusable products used in this study were conservative and may not match the actual lifespan. In our study we assumed instruments would last between 500 and 2000 times, based on estimates from replacement data from the DDUH. In another study the lifespan of all stainless steel products was defined as 3650 uses, which would reduce the overall environmental impact (Campion 2012). The distances transported were calculated based on the manufacturing location and the suggested transport routes using *Searates*. (www.searates.com) Some of the manufacturing locations were based on packaging information, which could be different from factory location. Additionally, all transport was calculated based on European transport LCA figures, not for example travel using Asian transport.

Electricity contributed 23.5% to the carbon footprint of an endodontic procedure.

Electricity can be harmful from an environmental perspective for a number of reasons. The LCA used electricity consumption values from the Ecoinvent database. These were based on estimates of Swedish electricity generation [30]. Most electricity production in Sweden comes from nuclear and hydroelectric power [31].

Traditionally the generation of electricity generally consumes significant amounts of water (power plants use a steam turbine to generate electricity, which also requires water for cooling [32].) Solar photovoltaic and wind power electricity production do not consume large quantities of water.

During an RCT procedure, the second largest contributor to GHG emissions (15.4%) was from the use of endodontic files. Traditionally root canal instruments have been considered multiple use, being discarded only when the operator visualised file damage or after a certain number of uses. Recently with the advent of new metal alloys (e.g. NiTi) manufacturer’s advice (ProTaper®, Dentsply-Sirona, Ballaigues, Switzerland) and legislation in the UK [33], the perception of repeated file use has been questioned or even contraindicated [34].

Although not adopted by all European countries, the inability to adequately clean root canal instruments (DOH 2005) has led to UK legislation demanding that files be discarded after single use, which has been supported by an increasing view that files should be considered as single use instruments for reasons of potential instrument fracture [35, 36]. Although this is not the current policy in Malmö University, who operate a limited single-use policy, it does highlight that this area of dentistry is likely to have increasing environmental impact in the future.

Within Malmö stainless steel endodontic K-files larger than ISO size 20 are used five times, while stainless steel K-files below ISO 20 and reciprocating NiTi Wave One Gold® files are disposed of after a single-use. During an RCT procedure it is necessary to begin by widening the root canal system in the coronal aspect with smaller files prior to progressing to larger stainless steel files or NiTi files. As a result, this has necessitated the use of a large number of stainless steel instruments in teaching and practice; however, recently there has been a significant effort by manufacturers and academics to reduce the number of files employed during RCT [37]. It is hoped that this trend will reduce waste as well as consider the impact on the environment, during the course of RCT in the future.

The packaging contributed 9% to the carbon footprint of the procedure. Of this packaging cardboard contributed close to 6% of the carbon footprint. Actual cardboard use may be different to the actual packaging of the products. Some items are additionally packaged in plastic bags (such as the evacuation tips) and in some cases large crates may be used. The materials and their total weight would subsequently modify the results in each of the impact categories.

Preparing the chair contributed 8% to the carbon footprint, of which the major contributor (7.6%) was isopropyl alcohol. There are alternatives perhaps that could be considered as alternatives to isopropyl alcohol, such as *Aloe Vera*-based products, essential oils (e.g. Propolis) and plant extracts [38] (Venkateshbabu et al. 2016), however this is outside the scope of this paper (e.g. McReynolds 2018 [39]). Further research is needed to propose other effective disinfectants that could perhaps replace isopropyl alcohol.

Soaps and detergents also contributed 9% to the carbon footprint. Depending on their make up they can be harmful to the environment, and clinicians should consider more environmentally friendly solutions which have less impact on eutrophication e.g. low phosphate detergents [40].

The paper used at the faculty comes from virgin pulp. This has a higher potential impact compared to sanitary paper from a recycled product [41]. Switching to sanitary paper which is sourced from recycled product would significantly reduce the carbon footprint of an endodontic procedure. To reduce the impact potential of sanitary paper within dentistry, other options must be considered. Substituting the use of paper towels during the hand washing process and replacing it with a warm air hand dryer could be one way to reduce the overall environmental impact of dental procedures although concerns with aerosol would need to be considered. The difference in using unbleached versus bleached sanitary paper or other more sustainable materials could be researched as other alternatives. An LCA assessment and patient safety assessment of the use of alcohol gel to reduce handwashing should also be considered [42].

Disposable bibs contributed around 7% to the carbon footprint of the endodontic procedure. The use of patient and operator bibs involve sanitary paper. The primary purpose of dental bibs are to protect the clothes of the health care workers and the patient from bodily and medicinal fluids that could potentially harm the individual or their clothing. Alternatives to these disposable bibs could be reusable bibs. Reusable dental bibs would need to be comfortable, durable and economical and would need to comply with government regulations. Previous studies have shown that reusable operating gowns are more sustainable compared to single-use disposable operating gowns while still meeting the needs of the health care sector [43, 44]., This would be applicable to dental bibs and are certainly a more sustainable alternative. Reducing the quantity of sanitary paper used would not just reduce CO_2_ eq emissions, but also the potential impact on acidification, marine eutrophication and terrestrial eutrophication.

Five percent of the CO_2_ eq release in an RCT procedure comes from the root canal sealer. Unlike an examination and a periodontal procedure, medicinal and botanical ingredients, including root canal sealers are an integral part of the RCT procedure and are necessary for achieving optimal results. The extent of which medicaments contribute to environmental damage once released into nature are not well known and requires further research [45], however, ongoing research into environmentally friendly alternatives continues with naturally sourced irrigants e.g. grape seed extract and antibacterial dressings e.g. propolis extract being investigated to replace current cytotoxic gold standards [46]. Furthermore, the advance and expansion of minimally invasive regenerative endodontics techniques designed to stimulate repair or regeneration of damaged pulp tissue using progenitor cell populations rather than simply replace the pulp, offers a future with natural biomimetic restorative solutions rather than current synthetic medicament-based solutions [47, 48].

In RCT procedures, the use of disposable plastic devices (e.g evacuation tips) was responsible for 10% of the environmental footprint. Decontamination documents such as the English HTM01–05 have supported the replacement of difficult to clean instruments such as root canal instruments, matrix bands, saliva ejectors, aspirator tips and three-in-one tips with single use items; however, this has environmental consequences. Alternatives made of stainless steel or a biodegradable material such as bamboo would be more sustainable but would need more research on the implications for patient safety prior to clinical introduction [49].

Endodontic consumables (e.g. gloves), as well as dentist and patient travel could be significantly reduced if the treatment was completed in one rather than the customary two visits. Although not recommended (or possible), in all instances there is increasing evidence to suggest that single visit can be as successful as multi-visit RCT if well carried out as well as being more cost-effective [50]. Furthermore, there are numerous other practical advantages to completing the endodontic treatment in one visit, including reduced recurring anxiety for patients, less postoperative pain, increased operator efficiency during chemo-mechanical debridement as well as obvious cost-effective advantages [49, 51]. The sustainability element of a one stage visit should be reinforced in teaching of RCT in the future at undergraduate level and completion of treatment, if possible, in one-visit encouraged.

Indeed, going one step further, perhaps another was to limit the environmental impact of RCT procedures, reduce travel, chair-time, endodontic product use as well as limiting the ongoing complexity of the restorative cycle [52], would be to avoid carrying out RCT in the first place. To that end, Endodontics is beginning to understand and embrace the role of vital pulp treatment procedures in limiting the destructive nature of RCT, with selective caries avoidance of pulp exposure and pulpotomy procedures being recommended in preference to traditional non-selective caries removal and pulp exposure or pulpectomy in cases of irreversible pulpitis [48, 53]. It is hoped, going forward that these minimally invasive, biologically base therapies will in turn reduce the environmental impact of dental procedures.

## Conclusion

The endodontic team need to consider how they can reduce the environmental burden of endodontic care. One immediate area of focus might be to consider environmentally friendly alternatives such as wind or solar generated alcohol, and alternatives to isopropyl alcohol including *Aloe Vera* and essential oils. Longer term, research into environmentally-friendly medicaments should continue to investigate the replacement of current cytotoxic gold standards with possible natural alternatives. A simple way for dentists to reduce the environmental impact of RCT would be to complete the treatment where possible in one visit, thereby reducing equipment, consumable costs for the dentist and travel costs for the patient. Finally, minimally invasive regenerative endodontics techniques designed to stimulate repair or regeneration of damaged pulp tissue may also be one way of improving the environmental impact of an RCT.

## Supplementary information

**Additional file 1**. Appendix 1.

**Additional file 2**. Appendix 2.

## Data Availability

The datasets generated during and/or analysed during the current study are not publicly available due to the fact they are using ecoinvent data sources.

## Abbreviations

CH4: Methane
CO2: Carbon Dioxide
CO_2_ eq emissions: Carbon Dioxide equivalent emissions
CTUe: Comparative Toxic Units ecotoxicity
CTUh: Comparative Toxic Unit for human
DALY: Disability-Adjusted life Years
DDUH: Dublin Dental University Hospital (DDUH)
Disease inc.: Disease incidence
EDTA: Ethylenediaminetetraacetic acid
FDI: Fédération Dentaire Internationale
GHG: Green House Gas(es)
GWP: Global Warming Potential
ISO: International Organization for Standardization (ISO)
kBq U235 eq: Kilogram Uranium 235 equivalent
kg CFC-11 eq: Kilogram equivalent of Chlorofluorocarbon 11
kg N eq: Kilogram equivalent of Nitrogen
kg NMVOC eq: Kilogram equivalent of Non-methane volatile organic compounds equivalent
kg P eq: Kilogram equivalent of Phosphorus
kg Sb eq: kilogram of Antimony (Sb) equivalents.
kW: Kilowatt
LCA: Life cycle assessment
LCIA: life cycle impact assessment
m3 depriv.: Cubic metre deprivation
MJ: Millijoules
molc H+ eq: moles of hydrogen equivalent
molc N eq: moles of nitrogen equivalent
NaOCl: Sodium hypochlorite solution (0.5–5%)
NiTi: Nickel Titanium
Pt: Production per unit of time
RCT: Root Canal Treatment
