# Supplementary material for: Environmental sustainability in endodontics. A life cycle assessment (LCA) of a root canal treatment procedure
Source: BMC Oral Health. 2020 Dec 1;20:348. doi: 10.1186/s12903-020-01337-7 (PMC7708105; doi:10.1186/s12903-020-01337-7)
Supplement: Supplementary file 1 — Additional file 1. Appendix 1. [file 12903_2020_1337_MOESM1_ESM.docx]

| **Item** | **Quantity** | **Material** | **Weight (g)** |
| --- | --- | --- | --- |
| Ca(OH)2 | 1 application | Ca(OH)2 | 0.666 |
| Cotton pellets | 12 | Cotton | 1.14 |
| EDTA | 5ml | 17% ((HO2CCH2)2NCH2CH2N(CH2CO2H)2) | 0.5 |
| Endoneedle for irrigation | 2 | Stainless Steel + Polyethylene | 1.934 |
| Evacuation tip | 2 | Polyethylene | 2.86 |
| Face mask | 4 | Non-woven fabric | 2.96 |
| Flexofile size 10 (25mm) | 2 | Stainless Steel | 0.17 |
| Flexofile size 15 (25mm) | 2 | Stainless Steel | 0.17 |
| Flexofile size 20 (25mm) | 2 | Stainless Steel | 0.17 |
| Flexofile size 25 (25mm) | 2 | Stainless Steel | 0.17 |
| Flexofile size 30 (35mm) | 2 | Stainless Steel | 0.18 |
| Foam for file holder | 1 | Polyether | 0.61 |
| Gloves | 8 | Nitrile | 7.1 |
| Gutta percha | 1 C point + 8 lateral | Rubber | 0.3 |
| Lidocaine anaesthesia | 2 | 1,8ml Inject + Glass cylinder | 5.65 |
| NaOCl Solution | 25ml | 2% NaOCl | 0.25 |
| Needle Anaesthesia | 2 | Stainless Steel + Polyethylene | 0.18 |
| Non-woven sponge big (guaze) | 4 | Non-woven fabric | 0.48 |
| Paper points | 18 | Virgin Pulp | 0.09 |
| Paper towel | 16 | Virgin Pulp | 2.3 |
| Patient bib | 2 | PE/Tissue | 7.58 |
| Plastic cup | 2 | Polypropylene | 2.46 |
| Plastic Sensor Cover | 6 | Polyethylene | 1.166 |
| ProTaper Gold F1 (25mm) | 2 | Nickel Titanium | 1.14 |
| ProTaper Gold F2 (25mm) | 2 | Nickel Titanium | 1.16 |
| ProTaper Gold F3 (25mm) | 2 | Nickel Titanium | 1.17 |
| ProTaper Gold S1 (25mm) | 2 | Nickel Titanium | 1.1 |
| ProTaper Gold S2 (25mm) | 2 | Nickel Titanium | 1.11 |
| ProTaper Gold Sx (17mm) | 2 | Nickel Titanium | 1.05 |
| Rubber dam | 2 | Latex | 4.54 |
| Small (micro) evacuation tip | 2 | Polypropylene & Polyethylene | 4.5 |
| Surgical aspirator tip | 2 | Polyethylene | 2.87 |
| Syringe 5ml | 4 | Polypropylene & Polyethylene | 5.92 |
| Syringe tip | 2 | Virgin Plastic | 0.83 |
| VIPS waste receiver | 2 | Polypropylene | 6.29 |
| Wipak steriking S20 | 1 | Plastic film and Kraft Paper | 10.96 |
| Wipak steriking S38 | 1 | Plastic film and Kraft Paper | 39.88 |
| ZOE filling | 1 filling | Zinc oxide (20g) + eugenol (25ml) | 1.1 |
