# Supplementary material for: Environmental sustainability in endodontics. A life cycle assessment (LCA) of a root canal treatment procedure
Source: BMC Oral Health. 2020 Dec 1;20:348. doi: 10.1186/s12903-020-01337-7 (PMC7708105; doi:10.1186/s12903-020-01337-7)
Supplement: Supplementary file 2 — Additional file 2. Appendix 2. [file 12903_2020_1337_MOESM2_ESM.docx]

| Item | Quantity | Material | Assumed Lifespan | Total Weight (g) |
| --- | --- | --- | --- | --- |
| Mirror handle | 2 | Stainless Steel/Silicone | 500 | 44.7 |
| Mirror head | 2 | Stainless Steel and glass | 50 | 13.26 |
| Dental Explorer | 2 | Stainless Steel/Silicone | 500 | 14.78 |
| Pocket probe | 2 | Stainless Steel | 500 | 17.47 |
| Straight/DG16 probe | 2 | Stainless Steel | 500 | 36.82 |
| College Forceps | 2 | Stainless Steel | 500 | 47.34 |
| Spoon excavator | 2 | Stainless Steel | 500 | 35.88 |
| Carver | 2 | Stainless Steel | 500 | 43.46 |
| Carver (small) | 1 | Stainless Steel | 500 | 21.17 |
| Flat plastic | 2 | Stainless Steel | 500 | 37.54 |
| Evacuation tip adaptor | 1 | Polypropylene | 500 | 3.31 |
| Small rectangular dish | 1 | Stainless Steel | 500 | 12.04 |
| Tray | 2 | Stainless Steel | 2000 | 658.12 |
| Rubber dam frame | 2 | Polypropylene | 500 | 7.95 |
| Clamp forceps | 1 | Stainless Steel | 500 | 72.99 |
| Bicuspid clamp | 1 | Stainless Steel | 500 | 1.54 |
| Molar clamp | 1 | Stainless Steel | 500 | 1.21 |
|  |  |  |  |  |
| Glass dappen dish | 2 | Glass | 500 | 73.96 |
| Endofile ruler | 1 | Polypropylene | 500 | 25.28 |
|  |  |  |  |  |
| Scissors | 1 | Stainless Steel | 500 | 18.16 |
| Gutta percha plugger | 1 | Stainless Steel | 500 | 21.46 |
| Long shank burs S | 1 | Stainless Steel | 5 | 0.63 |
| Extended burrs M | 1 | Stainless Steel | 5 | 0.65 |
|  |  |  |  |  |
| Endo file holder | 1 | Stainless Steel | 500 | 48.83 |
| Metal dappen dish | 3 | Stainless Steel | 500 | 33.6 |
| Small solution basin | 1 | Stainless Steel | 500 | 14.27 |
|  |  |  |  |  |
| Waste Fork | 1 | Stainless Steel | 500 | 5.32 |
| Tray Lid | 1 | Stainless Steel | 2000 | 463.24 |
| Spatula** | 1 | Stainless Steel | 500 | 21.59 |
| Glass tray** | 1 | Glass | 500 | 79.85 |
| File Stand | 1 | Stainless Steel | 500 | 28.41 |
| Endo Tabs | 1 | Polypropylene | 500 | 0.73 |
| High-speed hand piece | 1 | Electrical equipment | 2000 | 63.77 |
| Slow-speed hand piece | 1 | Electrical equipment | 2000 | 120.46 |
| X-smart +** | 1 | Electrical equipment | 2000 | 958.6 |
|  |  |  |  |  |
| Apex Locator** | 1 | Electrical equipment | 2000 | 136.79 |
| Planmeca Radiograph Sensor | 1 | Electrical equipment | 10000 | 33.85 |
| Insafe Injection** | 1 | Stainless Steel and PP,PE | 500 | 65.29 |
